# Lack of Reality: Positive Self-Perceptions of Health in the Presence of Disease

**DOI:** 10.3390/sports5020023

**Published:** 2017-04-06

**Authors:** Vincent J. Dalbo, Masaru Teramoto, Michael D. Roberts, Aaron T. Scanlan

**Affiliations:** 1 Human Exercise and Training Laboratory, Central Queensland University, Rockhampton, Queensland 4702, Australia; a.scanlan@cqu.edu.au; 2 Clinical Biochemistry Laboratory, Central Queensland University, Rockhampton, Queensland 4702, Australia; 3 Division of Physical Medicine and Rehabilitation, University of Utah, Salt Lake City, UT 84108, USA; Masaru.Teramoto@hsc.utah.edu; 4 Molecular and Applied Sciences Laboratory, Auburn University, Auburn, AL 36849, USA; mdr0024@auburn.edu

**Keywords:** obesity, diabetes, cardiovascular disease

## Abstract

The aim of this study was to determine if adults in Central Queensland have accurate self-perceptions of health. Data were collected as part of the 2010 Central Queensland Social Survey (N = 1289). Overweight/obesity is considered a health disorder and was determined using body mass index. Disease states were determined by asking respondents if they have: heart disease, high/low blood pressure, high cholesterol, high triglycerides, thyroid disorder, diabetes, and osteopenia/osteoporosis. Self-perceptions of health were assessed by asking, “Would you say that in general your health is” poor, fair, good, very good, excellent, don’t know, and no response. An accurate health perception occurred if: (1) A respondent with a disease/health disorder reported that their health was poor/fair or (2) A respondent without a disease/health disorder reported that their health was good/very good/excellent. The proportions of people with an accurate health perception by disease/health disorder were compared using a χ^2^ test. A proportion ratio (PR) with a 95% confidence interval (CI) was calculated for each disease/health disorder. A logistic regression analysis was performed to examine the association between each disease/health disorder and health perception using gender, age, education, physical activity level, and smoking status as covariates. More than 50% of residents with each disease/health disorder reported their health to be good/very good/excellent. Residents with each disease/health disorder were less likely to have an accurate health perception than those without the corresponding disease/health disorder prior to (*p* < 0.001) and following adjustment of the covariates (*p* < 0.001). Our results suggest that overweight/obesity and prevalence of disease are not being recognized as unhealthy, which contradicts established definitions of health.

## 1. Introduction

There is confusion regarding the definition of health amongst clinical researchers and the medical community which may be impacting the definition of health in the general population. However, there should be no confusion regarding the definition of health. The World Health Organization (WHO) defines health as a state of complete physical, mental, and social well-being [1], while Merriam-Webster defines health as being well or free of disease [2]. Using the established definitions of health an individual using medication to manage disease or someone who is overweight/obese is not healthy. 

Australians are living in an unhealthy society, evidenced by the high prevalence of overweight/obesity [3], increasing prevalence of disease [4,5], poor nutritional practices [6], low levels of physical activity [7], and low levels of exercise [8]. In turn, research has demonstrated health can be greatly improved with adherence to a proper diet [9] and implementation of exercise [10]. Moreover, Australians are aware of healthy eating practices [11] and know their health would be improved with exercise [12]. Despite this knowledge, Australians consume poor diets, engage in insufficient quantities of exercise, and appear to accept overweight/obesity and disease as a part of daily life. As a result, researchers have attempted to identify why Australians eat poor diets [13] and choose not to partake in sufficient levels of physical activity and exercise, yielding inconsistent findings [13,14]. Surprisingly, the only research found examining Australians’ perceptions of their own health, known as self-perceptions of health, occurred with data collected in 1981. The authors examined the association between self-perceptions of health and longevity in a random sample of older adults (≥60 years) residing in Sydney, Australia [15,16]. However, no found research has examined the accuracy of self-perceptions of health in the general population. 

Research examining self-perceptions of health is vital, as results may help explain why individuals choose to neglect their health. Specifically, if unhealthy individuals perceive themselves as healthy, they may lack motivation to alter nutritional, physical activity, and exercise habits to promote a healthier lifestyle. As a result, the aim of this investigation is to determine if residents of Central Queensland have accurate self-perceptions of health given their current health status. 

## 2. Materials and Methods

### 2.1. Pilot Testing

Prior to data collection, ethical clearance was granted by the Central Queensland University Human Research Ethics Committee (project number: H09/08-046; approval date: 2009). All procedures were conducted in accordance with the Declaration of Helsinki. Pilot testing on 50 randomly selected households was conducted to examine interview comments (e.g., inadequate response categories, confusing wording, question-order effects) and pre-test frequency distributions were reviewed to ensure questions did not require modification. The survey respondents were randomly drawn from a telephone database for Central Queensland, using a ten-station computer-assisted telephone interviewing system (Ci3 CATI System, Win Cati 4.2, Sawthooth Technologies; Northbrook, IL, USA) installed on a local area network. Following pilot testing, all questions were deemed appropriate for inclusion in the survey. Data obtained during pilot testing were not included as part of the survey results. 

### 2.2. Survey Methods

Data were collected in October and November 2010 as part of the Central Queensland Social Survey [8]. Central Queensland was defined as the area from the Mackay Statistical Division to the northern point of the Wide Bay Burnett Statistical Division, including Bundaberg. The region of Central Queensland was delineated into three areas for interviewing: Rockhampton Regional Council Area; Mackay Regional Council Area; and the Remainder of Central Queensland. A random selection process was employed to ensure respondents had an equal chance of being selected, using a two-stage process: (1) the selection of households; and (2) the selection of respondent gender in each household. One person was selected as the respondent for the interview per household. Respondents were 18 years or older and were living in a dwelling unit that could be contacted by a direct-dialed, land-based telephone service. If interviewers were unsuccessful establishing contact on their first call, a minimum of five callback attempts were made. In the event contact was not made following five callback attempts, the household was replaced. The average interview length was 29 min. Interview times complied with the revised Australian Communications and Media Authority Industry Standard for Research Calls.

### 2.3. Survey Respondents

Respondents completed a computer-assisted telephone interview representing 1289 completed interviews. The response rate of 36% is consistent with previous surveys from Central Queensland [17]. The Index of Dissimilarity demonstrated the sample collected during the survey varied from the target population according to census data [18] on the dimensions of geographic region and age. As a result, data were adjusted for sampling weights determined using a one-step multiple weighting for geographic location and age category, which has previously been reported [8]. 

### 2.4. Survey Structure

The survey consisted of a standardized introduction and questions related to demographic information, physical activity behaviors, disease states, and self-perceptions of health. Physical activity was assessed using a valid [19] and reliable [20] instrument based on the Active Australia Survey [21]. Disease states were determined by asking respondents if they have ever been told by a doctor that they have any of the following chronic health problems: heart disease, high/low blood pressure, high cholesterol, high triglycerides, thyroid disorder, diabetes, and osteopenia/osteoporosis. Overweight/obesity was determined using body mass index (BMI) ascertained by asking respondents to report their height and weight which we used to calculate BMI. We refer to overweight/obesity as a health disorder as the Australian Institute of Health and Welfare classifies obesity as a major risk factor for disease [22], but it should be noted that the American Medical Association classifies obesity as a disease [23]. Self-perceptions of health were assessed based on a valid and reliable question found in the Medical Outcomes Study [24]. Specifically, respondents were asked, “Would you say that in general your health is” with answer choices including poor, fair, good, very good, excellent, don’t know, and no response. 

### 2.5. Statistical Analyses

We defined that a respondent had an accurate perception of their health if: (1) they had a specific disease/health disorder at the time of the survey and responded that their health was poor/fair or (2) if they did not have a specific disease/health disorder at the time of the survey and responded that their health was good/very good/excellent. Proportions of those with an accurate health perception were calculated for each disease/health disorder. The proportions of people with an accurate health perception by disease/health disorder were compared using a χ^2^ test. Furthermore, a series of binary logistic regression analyses were performed to examine the association between each disease/health disorder and health perception using gender, age, education, physical activity level, and smoking status as covariates. The covariates were selected because of their known association to health status. As per the guidelines of the Active Australia Survey [25], sufficient physical activity was defined as the accumulation of at least 150 min of activity and at least five sessions of activity over one week. Smoking status was determined by asking respondents if they were presently a smoker, as defined as smoking at least one cigarette per day for the past month. Statistical significance for all tests was set a priori at *p* < 0.05. All statistical analyses were conducted using IBM SPSS Statistics (v 22.0, IBM Corporation, Armonk, NY, USA). 

## 3. Results

Descriptive information of respondents can be found in Table 1. Surprisingly, 85.4% of residents who were overweight and 71.1% of those who were obese reported that their health was good/very good/excellent, while greater than 50% of residents with heart disease (51.6%), high/low blood pressure (70.6%), high cholesterol (73.7%), high triglycerides (74.4%), thyroid disorder (72.9%), diabetes (58.0%), and osteopenia/osteoporosis (62.5%) reported that their health was good/very good/excellent. Furthermore, residents with each disease/health disorder were less likely to have an accurate health perception than those without the corresponding disease/health disorder. For instance, there were a significantly lower proportion of residents who were overweight (χ^2^ = 459.37, *p* < 0.001) or obese (χ^2^ = 263.25, *p* < 0.001) who had an accurate perception of health in comparison to residents who were normal weight. Specifically, 14.6% of overweight residents and 28.9% of obese residents believed their health was poor/fair (=accurate health perception), while 88.3% of residents who were normal weight believed their health was good/very good/excellent (=accurate health perception). The disease/health disorder associated with the smallest proportion of residents with an accurate perception of health was overweight (14.6%); however, in all instances less than 50.0% of residents with disease reported their health was poor/fair: heart disease (48.4%), high/low blood pressure (29.4%), high cholesterol (26.3%), high triglycerides (25.6%), thyroid disorder (27.1%), diabetes (42.0%), and osteopenia/osteoporosis (37.5%). These results indicate a small proportion of residents with a disease/health disorder believe their health was poor/fair (Table 2). 

The results of the binary logistic regression analyses are summarized in Table 3. After adjusting for gender, age, education, physical activity level, and smoking status, residents with any disease/health disorder were significantly less likely to have an accurate perception of their health than those without the corresponding disease/health disorder (*p* < 0.001). For example, the odds of having an accurate perception of health for overweight and obese residents were 0.01 (95% CI = 0.01–0.02) and 0.03 (95% CI = 0.02–0.05) times the odds for normal weight residents, respectively.

## 4. Discussion

Results from our investigation highlight that residents of Central Queensland have misperceptions of health according to standard definitions of health [1,2]. The apparent disconnect in the understanding of health is concerning, as disease prevalence is on the rise [4] and a contributing factor may be individuals who are at risk for disease or have been diagnosed with a disease perceive themselves to be healthy and as a result do not comprehend the need to modify lifestyle factors that would improve health, such as the implementation of a healthier diet and exercise. 

One of the most significant issues concerning the health of Australians is overweight/obesity due to the relationship with an assortment of comorbidities including those examined in our investigation: heart disease, high/low blood pressure, high cholesterol, high triglycerides, thyroid disorder, diabetes, and osteopenia/osteoporosis [26], yet 85.4% of overweight residents and 71.1% of obese residents reported themselves to be healthy. Furthermore, heart disease is the leading cause of death in Australia [4] but 51.6% of residents with heart disease classified themselves as healthy. In turn, the majority of individuals with disease(s) that are classified as risk factors for heart disease perceive themselves as healthy. Specifically, 70.6% with high/low blood pressure, 73.7% with high cholesterol, 74.4% with high triglycerides, and 72.9% with thyroid disorder classified themselves as healthy. Diabetes is the fastest growing chronic disease in Australia [27] representing a significant financial cost to the government [27] and loss of life to the individual [28], yet 58.0% of residents with diabetes classify themselves as healthy. Those with osteopenia/osteoporosis follow the same trend as 62.5% classify themselves as healthy. 

Research from Canada [29], the United Kingdom [30], and Mexico [31] suggest a large proportion of adults have inaccurate self-perceptions of their weight status. Of particular interest were the findings from the United Kingdom in which 30.9% of females and 54.7% of males who were classified as overweight according to BMI measures obtained by a trained researcher, identified themselves as having a body weight that was “about the right weight” rather than “too heavy” or “too light” [30]. The investigation conducted in Mexico also produced concerning results. Specifically, respondents to the Mexican National Health and Nutrition Survey 2012 were asked their height and weight to be classified as normal weight, overweight, or obese according to BMI. Only 6.1% of non-diagnosed obese respondents correctly identified themselves as obese, while 8.8% of obese respondents who were diagnosed by a medical professional within the last 12 months as being obese correctly identified themselves as obese [31]. Results from these investigations [29,30,31] provide evidence suggesting a large proportion adults have inaccurate self-perceptions of their own weight status.

In the United States, Tarasenko et al. [32] examined differences in perceptions of childhood (8–15 years) overweight/obesity with respect to the children, their guardians, and their health care providers. Results from the study found misperceptions of weight status were abundant, as over 30% of physicians, and over 50% of overweight/obese children and their guardians had misperceptions of the weight status of the child. Moreover, misperceptions of the weight status were 6.3% greater in rural physicians, 11.3% greater in rural children, and 6.0% greater in rural adults of overweight/obese children compared to urban counterparts. Likewise, recall of health care providers informing guardians their child was overweight/obese was 6.3% lower in rural compared to urban environments. The experimenters also found children with misperceived overweight/obesity status were less likely to engage in weight control behaviors than children with accurate perceptions of overweight/obesity status.

The work from Tarasenko et al. [32] is important for several reasons. First, our data were collected in a regional population and it would be of interest for future research to examine if residents in urban environments have the same misperceptions of their own health. Second, we believe future research should explore the effects of self-perceptions of health on willingness to change behavior. Finally, our results suggest the average Central Queensland resident is unhealthy to the point that overweight/obesity and prevalence of disease are not recognized as being harmful. We also postulate physicians may not be informing patients who are overweight/obese or controlling disease with medication that they are unhealthy. Tarasenko et al. [32] theorized the greater misperceptions of health in rural environments was possibly due to the historically higher prevalence of overweight/obesity in rural children, which may have resulted in a norm shift where children, guardians, and health care providers are less likely to perceive children as overweight/obese because in comparison to their peers they are normal weight. This hypothesis may also be relevant in our study, as 60.3% of residents were overweight/obese while 85.4% of overweight residents and 71.1% of obese residents reported their health to be good/very good/excellent. There was a similar trend for each disease state in which more than 50% of residents with heart disease, high/low blood pressure, high cholesterol, high triglycerides, thyroid disorder, diabetes, and osteopenia/osteoporosis classified their health as good/very good/excellent. 

It is apparent that Australia has become an unhealthy society with 63% of adult residents being overweight/obese and 25% of children being overweight/obese [4]. Furthermore, overweight/obesity and prevalence of disease in Australia continues to rise [4]. Government initiatives are in place to encourage healthy eating and the promotion of physical activity, but it can be argued that past initiatives have had little success due to the rising rates of overweight/obesity, prevalence of disease, and increased health care expenditure [4]. While it is not the aim of this investigation to examine the efficacy of public health initiatives, our results suggest residents of Central Queensland may not have the desire to alter their lifestyle to improve health, as they currently view themselves as healthy. As a result, if we want to improve the health of Australians we likely need to change the current perception of health. Our work has established a misunderstanding of health exists amongst Central Queensland residents, but further research needs to determine why the misconceptions about health exist and if there is a cause and effect relationship between having a correct perception of health and being physically healthy.

## 5. Conclusions 

Central Queensland residents have misconceptions about health. The average resident of Central Queensland appears to be unhealthy to the point that overweight/obesity and prevalence of disease are not recognized as being harmful, which contradicts the established definitions of health [1,2]. The general health of Central Queensland residents must improve. Public health initiatives are consistently being developed and implemented in Australia, but the prevalence of overweight/obesity [3] and disease [5] continues to increase. Results from our investigation suggest Central Queensland residents may not have the desire to alter their lifestyle to improve health as they currently view themselves as healthy. 

## Figures and Tables

**Table 1:** Descriptive information of survey respondents.

Variable	Categories	Frequency (%)
Gender	Male	644 (49.7)
	Female	653 (50.3)
Age	18–34 years	400 (30.9)
	35–44 years	254 (19.6)
	45–54 years	236 (18.2)
	55+ years	407 (31.4)
Body Mass Index	<18.5 kg/m^2^	22 (1.7)
	18.5–24.9 kg/m^2^	392 (30.2)
	25.0–29.9 kg/m^2^	459 (35.4)
	≥30 kg/m^2^	322 (24.9)
	No response	102 (7.8)
Household Income (AUD)	Up to $26,000	162 (12.5)
	$26,001–$52,000	149 (11.5)
	$52,001–$100,000	233 (17.9)
	>$100,000	336 (25.9)
	Don’t know/No response	417 (32.2)
Years of Education	1–10 years	371 (28.6)
	11–12 years	335 (25.9)
	13–14 years	209 (16.1)
	15+ years	375 (28.9)
	No schooling	1 (0.1)
	Don’t know/No response	5 (0.4)
Sufficient Physical Activity	Yes	573 (55.0%)
	No	470 (45.0%)
Smoking Status	Yes	227 (17.5%)
	No	1070 (82.5%)

Notes: N = 1289; data were adjusted for sampling weights. AUD is the Australian Dollar. As per the guidelines of the Active Australia Survey [25], sufficient physical activity was defined as the accumulation of at least 150 min of activity and at least five sessions of activity over one week. Smoking status was determined by asking respondents if they were presently a smoker, as defined as smoking at least one cigarette per day for the past month.

**Table 2:** Self-perceptions of health by disease/health disorder in rural and regional Central Queensland residents.

Condition	Presence or Absence of Disease/Disorder	Perception of Health (%)		χ^2^	*p*
		Correct	Incorrect		
**Health Disorder**					
Overweight ††	Yes (53.9%)	14.6%	85.4%	459.37	<0.001 †
	No (46.1%)	88.3%	11.7%		
Obese †††	Yes (45.1%)	28.9%	71.1%	263.25	<0.001 †
	No (54.9%)	88.3%	11.7%		
**Disease**					
Heart disease	Yes (7.3%)	48.4%	51.6%	81.26	<0.001 †
	No (92.7%)	85.0%	15.0%		
High/low blood pressure	Yes (23.4%)	29.4%	70.6%	38.71	<0.001 †
	No (76.6%)	86.3%	13.7%		
High cholesterol	Yes (16.7%)	26.3%	73.7%	323.32	<0.001 †
	No (83.3%)	84.4%	15.6%		
High triglycerides	Yes (3.3%)	25.6%	74.4%	88.98	<0.001 †
	No (96.7%)	82.9%	17.1%		
Thyroid disorder	Yes (4.6%)	27.1%	72.9%	112.69	<0.001 †
	No (95.4%)	83.1%	16.9%		
Diabetes	Yes (6.8%)	42.0%	58.0%	98.00	<0.001 †
	No (93.2%)	84.4%	15.6%		
Osteopenia/Osteoporosis	Yes (4.9%)	37.5%	62.5%	85.73	<0.001 †
	No (95.1%)	83.7%	16.3%		

Notes: An accurate perception of health was defined by having a disease/health disorder and responding that health was poor/fair or by not having a disease/ health disorder and responding that health was good/very good/excellent. The χ^2^ test was used to compare the proportions of residents with an accurate health perception by disease/health disorder. † = Residents with a disease/health disorder were significantly less likely to have an accurate perception of health than residents without a disease/health disorder. ††: Excluding obese and underweight residents. †††: Excluding overweight and underweight residents.

**Table 3:** Results of binary logistic regression analyses on perception of health.

Condition	B (SE)	*p*	OR (95% CI)
**Health Disorder**			
Overweight ††	−4.48 (0.27)	<0.001	0.01 (0.01–0.02) †
Obese †††	−3.50 (0.26)	<0.001	0.03 (0.02–0.05) †
**Disease**			
Heart disease	−1.87 (0.28)	<0.001	0.16 (0.09–0.27) †
High/low blood pressure	−2.92 (0.21)	<0.001	0.05 (0.04–0.08) †
High cholesterol	−2.94 (0.22)	<0.001	0.05 (0.04–0.08) †
High triglycerides	−2.75 (0.43)	<0.001	0.06 (0.03–0.15) †
Thyroid disorder	−2.99 (0.39)	<0.001	0.05 (0.02–0.11) †
Diabetes	−2.03 (0.29)	<0.001	0.13 (0.07–0.23) †
Osteopenia/Osteoporosis	−2.48 (0.37)	<0.001	0.08 (0.04–0.17) †

Notes: A model was built separately for each disease/health disorder. Each model was adjusted by gender, age, education, physical activity level, and smoking status. The outcome variable was perception of health coded as 0 = incorrect perception and 1 = correct perception. Each disease/health disorder variable was a dichotomous variable with the presence of a disease/health disorder as a reference category. †: Residents with a disease/health disorder were significantly less likely to have an accurate perception of health than residents without a disease/health disorder. ††: Excluding obese and underweight residents. †††: Excluding overweight and underweight residents.
